# Supplementary figures and images for: DFMG decreases angiogenesis to uphold plaque stability by inhibiting the TLR4/VEGF pathway in mice
Source: PLoS One. 2024 Apr 18;19(4):e0302387. doi: 10.1371/journal.pone.0302387 (PMC11025810; doi:10.1371/journal.pone.0302387)

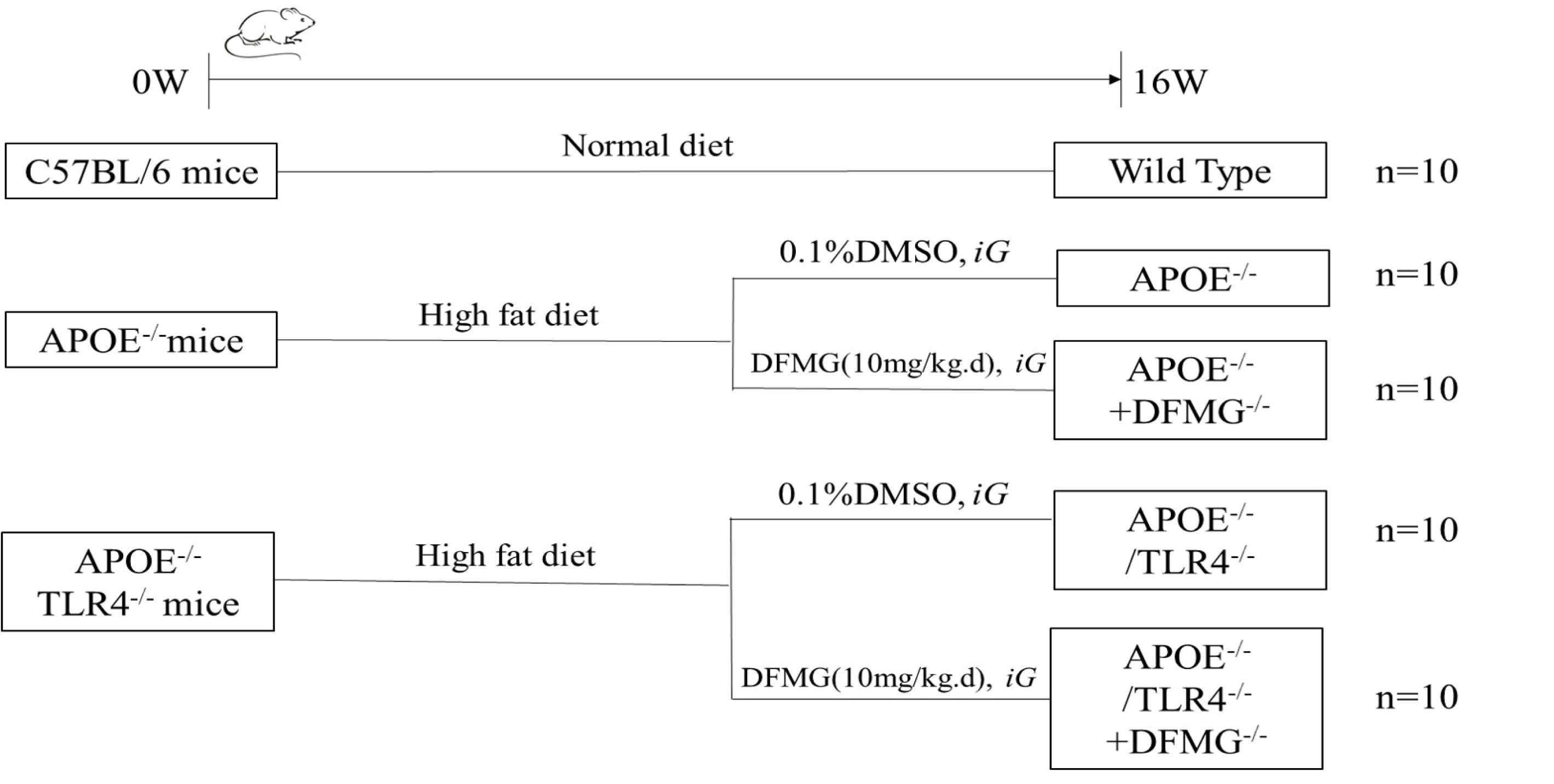

Supplement: S1 Fig — iG: intragastric infusion. (TIF) [file pone.0302387.s001.tif]

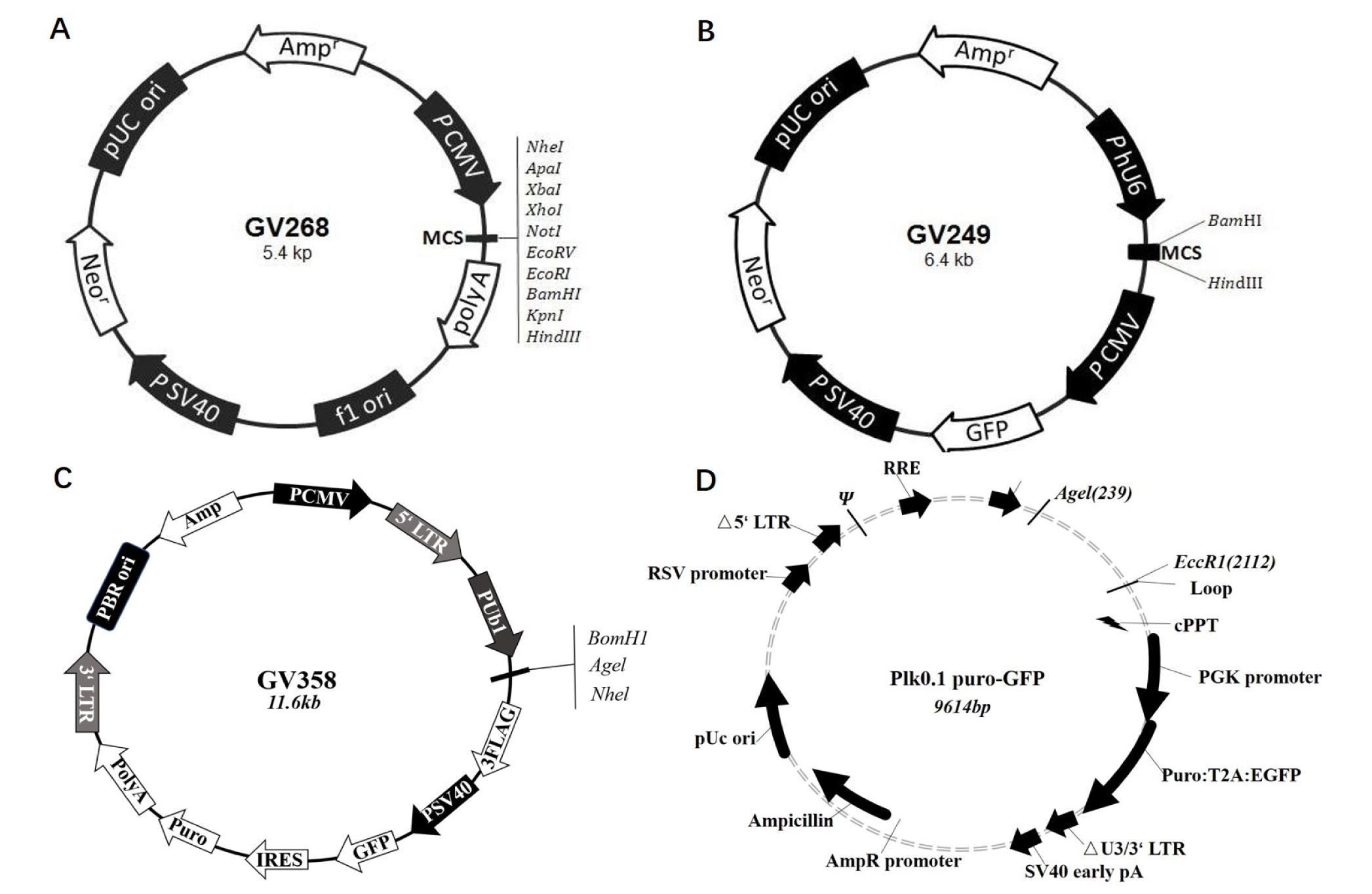

Supplement: S2 Fig — (A:GV268, B:GV249, C: GV358, D: Plko.1 puro-GFP). (TIF) [file pone.0302387.s002.tif]

Fig 1E

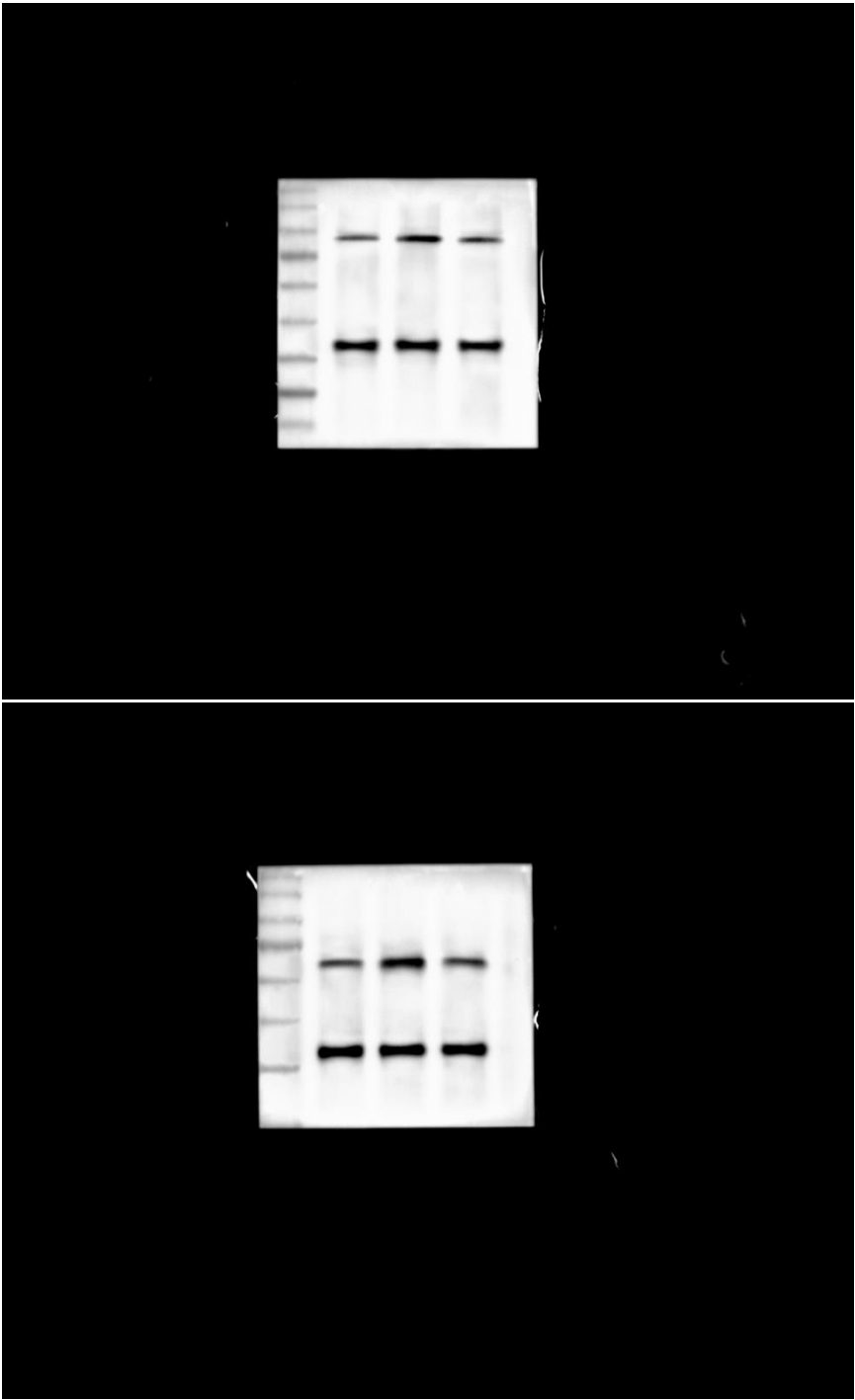

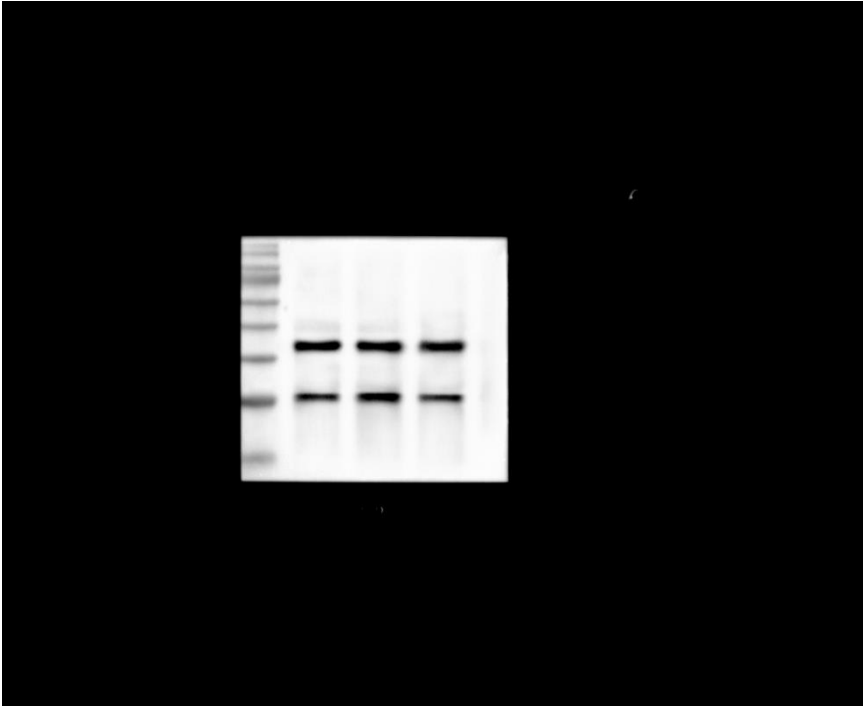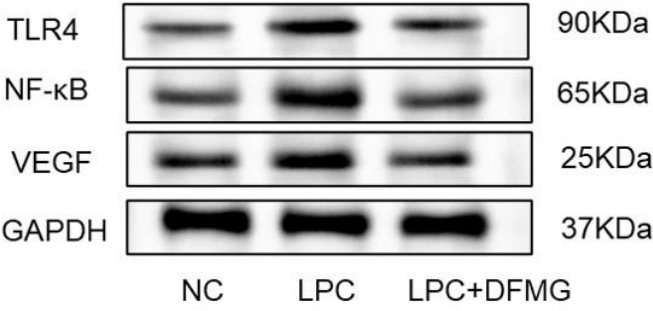

Fig 2B

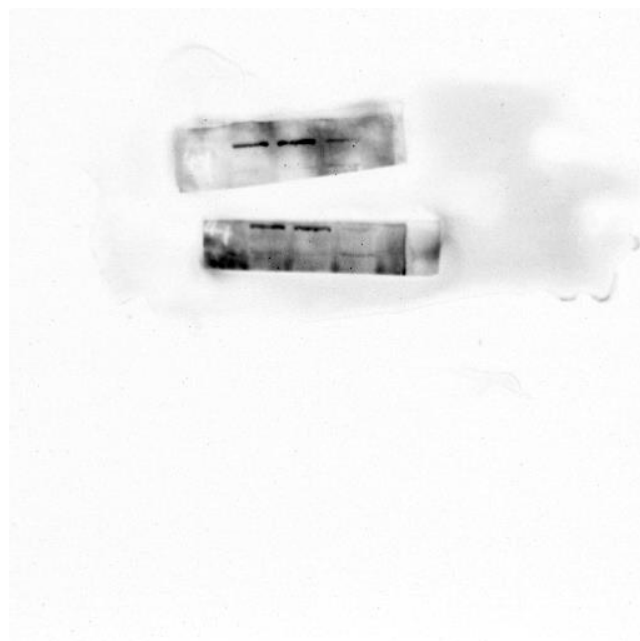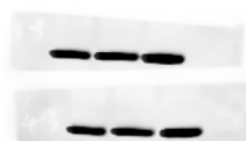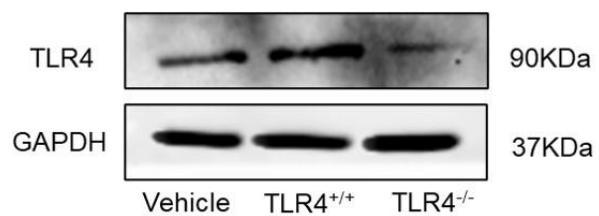

Fig 3B

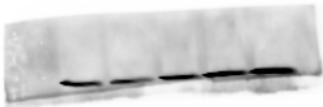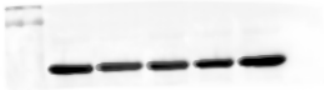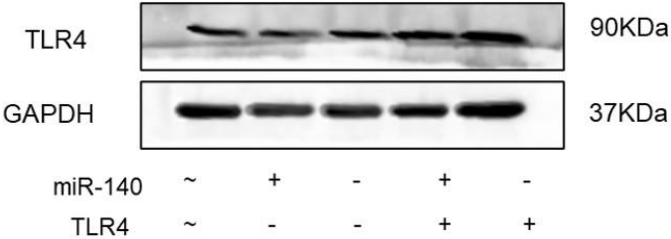

Fig 4A

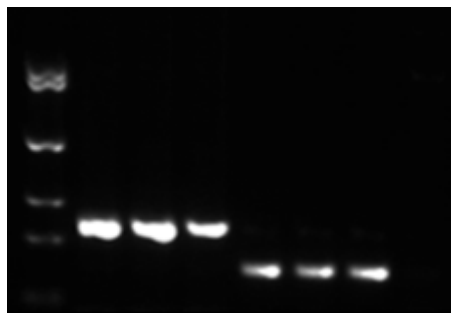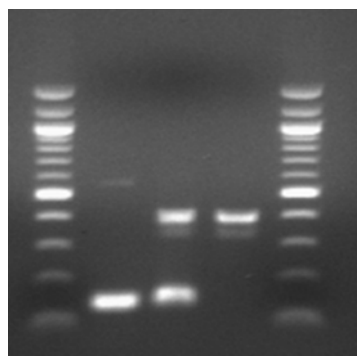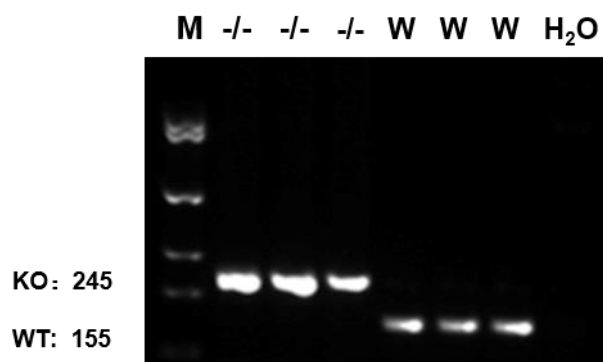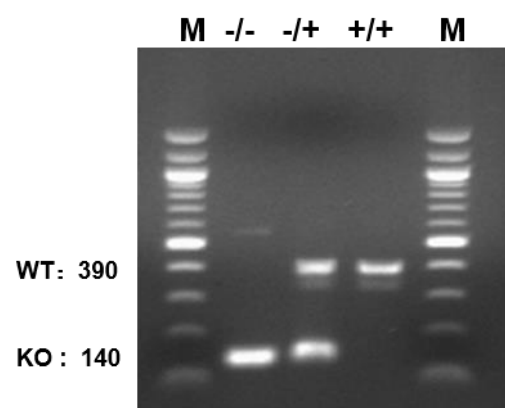

Fig 5 A

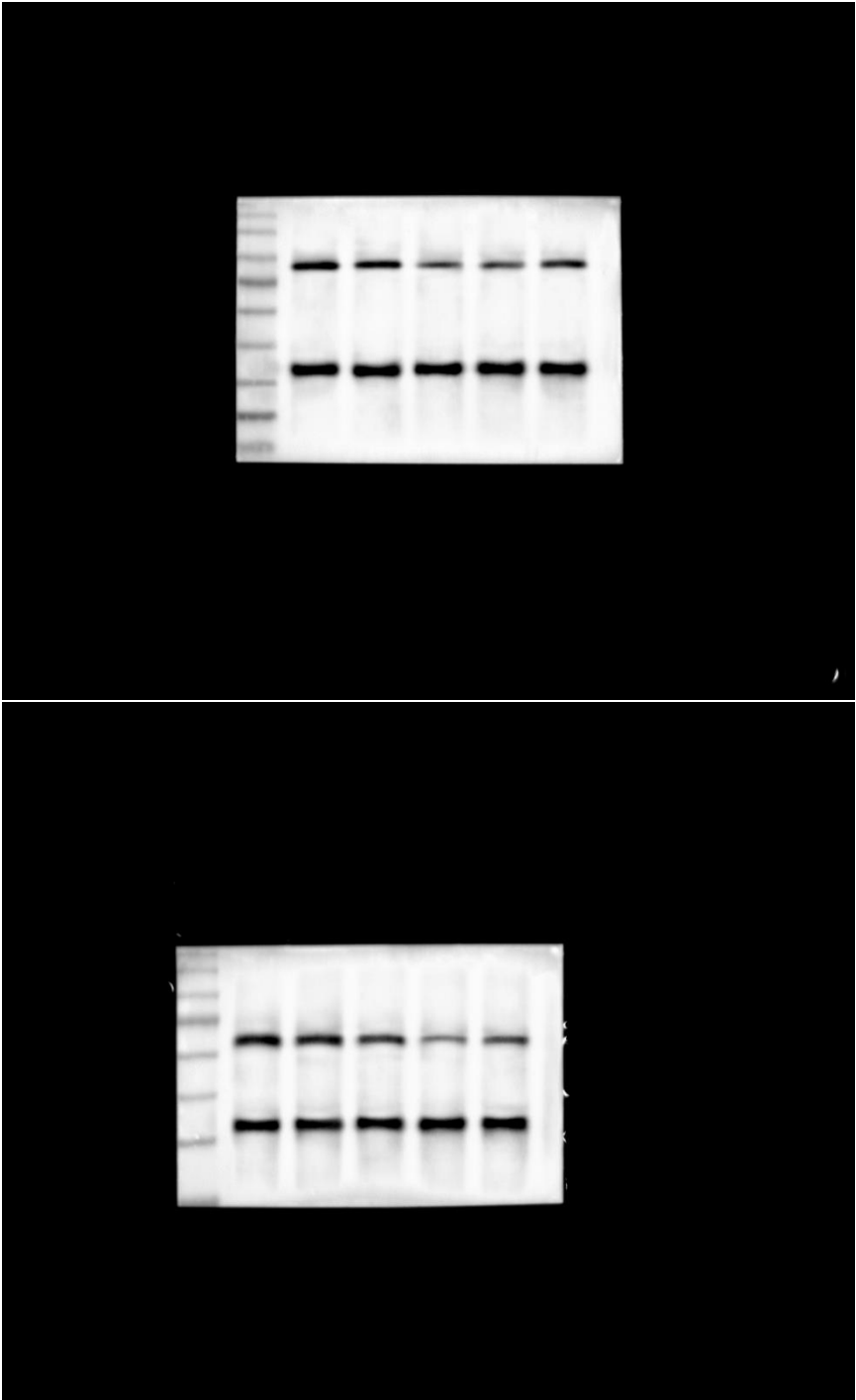

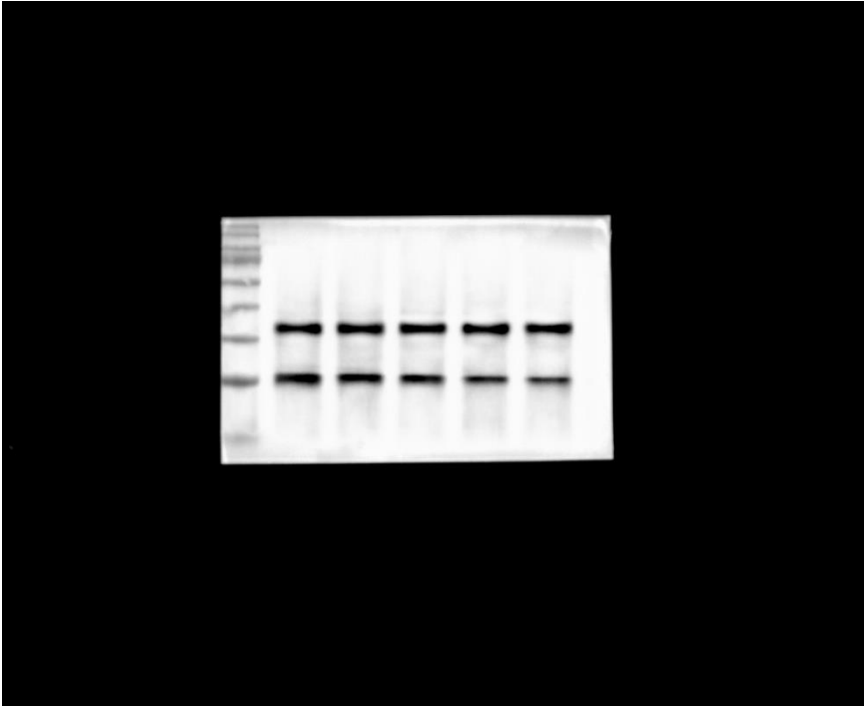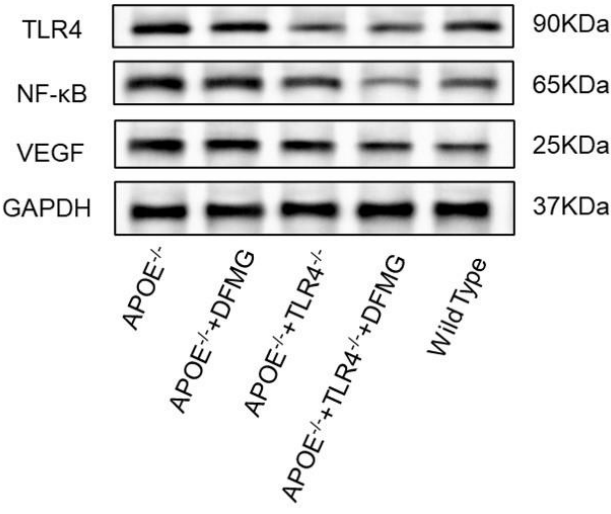

Supplement: S1 Raw images — (PDF) [file pone.0302387.s003.pdf]
