# Supplementary material for: DFMG decreases angiogenesis to uphold plaque stability by inhibiting the TLR4/VEGF pathway in mice
Source: PLoS One. 2024 Apr 18;19(4):e0302387. doi: 10.1371/journal.pone.0302387 (PMC11025810; doi:10.1371/journal.pone.0302387)
Supplement: Attachment — Submitted filename: Response to Editor.docx [file pone.0302387.s005.docx]

Dear Arvin Haj-Mirzaian,

Thank you for your prompt and constructive feedback on my manuscript entitled " DFMG may potentially decrease angiogenesis to preserve plaque stability by inhibiting the TLR4/VEGF pathway in atherosclerosis", which I submitted to PLOS ONE. I appreciate the time and effort you and the reviewers have invested in evaluating my work.

I have carefully considered each of your comments and made the necessary revisions to address the concerns raised. Below, I outline the changes made in response to your suggestions:

1. Please ensure that your manuscript meets PLOS ONE's style requirements, including those for file naming. The PLOS ONE style templates can be found at https://journals.plos.org/plosone/s/file?id=wjVg/PLOSOne_formatting_sample_main_body.pdf and https://journals.plos.org/plosone/s/file?id=ba62/PLOSOne_formatting_sample_title_authors_affiliations.pdf

Response 1: I have made revisions according to the formatting requirements of the PLOS ONE journal.

2. Note from Emily Chenette, Editor in Chief of PLOS ONE, and Iain Hrynaszkiewicz, Director of Open Research Solutions at PLOS: Did you know that depositing data in a repository is associated with up to a 25% citation advantage (https://doi.org/10.1371/journal.pone.0230416)? If you’ve not already done so, consider depositing your raw data in a repository to ensure your work is read, appreciated and cited by the largest possible audience. You’ll also earn an Accessible Data icon on your published paper if you deposit your data in any participating repository (https://plos.org/open-science/open-data/#accessible-data).

Response 2: Thank you for your valuable advice. However, I have not yet uploaded the raw experimental data to the repository at the moment.

3. Thank you for stating the following financial disclosure:

" This study is supported by the Natural Science Foundation of China (grant. no. 81370382), and Natural Science Foundation of Hunan Province (grant. no. 14JJ2059 and 2022JJ30415). ZY and LLS are authors who received awards."

Please state what role the funders took in the study. If the funders had no role, please state: ""The funders had no role in study design, data collection and analysis, decision to publish, or preparation of the manuscript.""

If this statement is not correct you must amend it as needed.

Please include this amended Role of Funder statement in your cover letter; we will change the online submission form on your behalf.

Response 3: This study is supported by the Natural Science Foundation of China (Grant No. 81370382) and the Natural Science Foundation of Hunan Province (Grant No. 14JJ2059 and 2022JJ30415). Xiaohua Fu and Yong Zhang, recipients of awards. Their contribution to this article involved reviewing and editing.

4. Please include your full ethics statement in the ‘Methods’ section of your manuscript file. In your statement, please include the full name of the IRB or ethics committee who approved or waived your study, as well as whether or not you obtained informed written or verbal consent. If consent was waived for your study, please include this information in your statement as well.

Response 4: We have incorporated an ethical statement and supplied the complete name and code of the Ethics Committee for the "mice" section in the Methods.

5. PLOS ONE now requires that authors provide the original uncropped and unadjusted images underlying all blot or gel results reported in a submission’s figures or Supporting Information files. This policy and the journal’s other requirements for blot/gel reporting and figure preparation are described in detail at https://journals.plos.org/plosone/s/figures#loc-blot-and-gel-reporting-requirements and https://journals.plos.org/plosone/s/figures#loc-preparing-figures-from-image-files. When you submit your revised manuscript, please ensure that your figures adhere fully to these guidelines and provide the original underlying images for all blot or gel data reported in your submission. See the following link for instructions on providing the original image data: https://journals.plos.org/plosone/s/figures#loc-original-images-for-blots-and-gels.

In your cover letter, please note whether your blot/gel image data are in Supporting Information or posted at a public data repository, provide the repository URL if relevant, and provide specific details as to which raw blot/gel images, if any, are not available. Email us at plosone@plos.org if you have any questions.

Response 5: The blot/gel images in this article have been uploaded in the required PDF format as requested.

6. We notice that your supplementary figures are uploaded with the file type 'Figure'. Please amend the file type to 'Supporting Information'. Please ensure that each Supporting Information file has a legend listed in the manuscript after the references list

Response 6: We have made revisions and re-uploaded.

The following are comments in response to other editors:

All procedures involving animals were conducted in strict accordance with ARRIVE guidelines for the care and use of laboratory animals and were approved by the Ethics Committee of Hunan Normal University (Approval No. 2013289). Researchers engaged in animal experimentation possess the Laboratory Animal Professional Post qualification, verified by the certificate code 20180054.

The -7-week-old SPF-grade ApoE gene knockout mice and the 6-7-week-old SPF-grade TLR4 gene knockout mice were graciously donated by the Collaborative Innovation Center for Model Animal at Wuhan University (Animal Qualification Testing Report Number: BJYRL-WBKH-20160224A1). The ApoE and TLR4 double gene knockout mice were bred by our research team through the mating of the aforementioned gene knockout mice. Non-transgenic mice with the same genetic background, aged 5-6 weeks (SPF-grade C57 BL/6 mice), were procured from Hunan Slake Company. All experimental mice were housed in the SPF-grade animal facility at the School of Medicine, Hunan Normal University, maintaining a 12-hour light/12-hour dark cycle. More detailed experimental methods and schematic diagrams can be found in the Methods section of the manuscript and in the S1 figures.

Conflict of Interest: The authors declare that they have no known competing financial interests or personal relationships that could have appeared to influence the work reported in this paper.

I believe these revisions have strengthened the manuscript, improving its overall clarity and coherence. I trust that these changes align with the expectations of PLOS ONE and enhance the quality of the manuscript.

Please find the revised manuscript attached. I hope these modifications meet the standards of the journal. If you require any further clarification or additional information, please do not hesitate to contact me.

Thank you once again for your valuable guidance throughout this process. I look forward to hearing from you soon.

Sincerely,

The Authors: Pingjuan Bai
