# Supplementary material for: DFMG decreases angiogenesis to uphold plaque stability by inhibiting the TLR4/VEGF pathway in mice
Source: PLoS One. 2024 Apr 18;19(4):e0302387. doi: 10.1371/journal.pone.0302387 (PMC11025810; doi:10.1371/journal.pone.0302387)
Supplement: Attachment — Submitted filename: Response to Editor.docx [file pone.0302387.s006.docx]

Dear Arvin Haj-Mirzaian,

We would like to express our sincere gratitude to you and the reviewers for the constructive feedback and valuable suggestions provided for our manuscript entitled " DFMG decreases angiogenesis to uphold plaque stability by inhibiting the TLR4/VEGF pathway in mice"[ PONE-D-23-29456R1]. We have carefully considered all the comments and suggestions provided by the reviewers and have made the necessary revisions to improve the quality and clarity of the manuscript.

*Journal Requirements:*

*Please review your reference list to ensure that it is complete and correct. If you have cited papers that have been retracted, please include the rationale for doing so in the manuscript text, or remove these references and replace them with relevant current references. Any changes to the reference list should be mentioned in the rebuttal letter that accompanies your revised manuscript. If you need to cite a retracted article, indicate the article&rsquo;s retracted status in the References list and also include a citation and full reference for the retraction notice.*

Response：

After a thorough examination of the references section of our paper, we confirm that no citations of retracted papers were found. Furthermore, the format of the references adheres to the requirements of your esteemed journal.

We believe that these revisions have strengthened the manuscript and addressed the concerns raised by the reviewers. We hope that these changes meet the expectations of the journal and contribute to the improvement of the manuscript.

Thank you once again for the opportunity to revise and resubmit our manuscript. We look forward to hearing from you regarding the further processing of our submission.

Sincerely,

Pingjuan Bai
